# Comparison of the Phytochemical Composition of *Serenoa repens* Extracts by a Multiplexed Metabolomic Approach

**DOI:** 10.3390/molecules24122208

**Published:** 2019-06-13

**Authors:** Guillaume Marti, Philippe Joulia, Aurélien Amiel, Bernard Fabre, Bruno David, Nicolas Fabre, Christel Fiorini-Puybaret

**Affiliations:** 1 UMR 152 Pharma Dev, Université de Toulouse, IRD, UPS, 31400 Toulouse, France; guillaume.marti@univ-tlse3.fr (G.M.); aurelien.amiel@yahoo.fr (A.A.); nicolas.fabre@univ-tlse3.fr (N.F.); 2 Institut de Recherche Pierre Fabre, Centre de R&D Pierre Fabre, Green Mission Pierre Fabre, 3 Avenue Hubert Curien, BP 13562, 31035 Toulouse CEDEX, France; philippe.joulia@pierre-fabre.com (P.J.); bernard.fabre@pierre-fabre.com (B.F.); bruno.david@pierre-fabre.com (B.D.)

**Keywords:** metabolomics, phytochemical equivalence, *Serenoa repens* extract, Saw palmetto, natural products

## Abstract

Phytochemical extracts are highly complex chemical mixtures. In the context of an increasing demand for phytopharmaceuticals, assessment of the phytochemical equivalence of extraction procedures is of utmost importance. Compared to routine analytical methods, comprehensive metabolite profiling has pushed forward the concept of phytochemical equivalence. In this study, an untargeted metabolomic approach was used to cross-compare four marketed extracts from *Serenoa repens* obtained with three different extraction processes: ethanolic, hexanic and sCO_2_ (supercritical carbon dioxide). Our approach involved a biphasic extraction of native compounds followed by liquid chromatography coupled to a high-resolution mass spectrometry based metabolomic workflow. Our results showed significant differences in the contents of major and minor compounds according to the extraction solvent used. The analyses showed that ethanolic extracts were supplemented in phosphoglycerides and polyphenols, hexanic extracts had higher amounts of free fatty acids and minor compounds, and sCO_2_ samples contained more glycerides. The discriminant model in this study could predict the extraction solvent used in commercial samples and highlighted the specific biomarkers of each process. This metabolomic survey allowed the authors to assess the phytochemical content of extracts and finished products of *S. repens* and unequivocally established that sCO_2_, hexanic and ethanolic extracts are not chemically equivalent and are therefore unlikely to be pharmacologically equivalent.

## 1. Introduction

Phytochemical extracts are complex mixtures containing dozens to thousands of compounds with a large spectrum of physico-chemical properties. Unlike synthetic drugs, the quality of herbal products can vary depending on the botanical species, geographical origin, growing and harvesting conditions and other parameters, like the choice of the solvent, extraction process and formulation [1,2]. Moreover, pre and post-extraction treatments can also impact the final composition of a marketed product [3]. Usually, the assessment of the overall quality of phytochemical extracts is centered on the quantitative and/or qualitative analysis of a few biomarkers, due to a lack of purified standards and the difficulty to implement straightforward multi-target analytical methods [4]. In incidences where active ingredients are not well characterized, the World Health Organization (WHO) introduced the notion of chromatographic fingerprints to assess the phytochemical composition of herbal products [5]. According to the WHO, since an herbal substance or herbal preparation in its entirety is regarded as the active substance, a comprehensive determination of the stability of the constituents with known therapeutic activity should also be demonstrated, by means of appropriate fingerprint chromatograms. This concept has been implemented in several guidelines published by regulatory agencies, such as the Food and Drug Administration (FDA) or the European Medicines Agency (EMA) [6,7]. The scientific community has developed several methods to generate phytochemical chromatographic fingerprints including comparative methods through several analytical platforms, such as one-dimensional nuclear magnetic resonance (1D-NMR) [8] and infrared spectroscopy [9], and separative methods based on high-performance liquid chromatography (HPLC) hyphenated to ultraviolet detection [10] or high-resolution mass spectrometry (HRMS) [11]. However, these methodologies do not necessarily involve a thorough identification of all components *in mixturae* and are limited to comparisons of spectroscopic or chromatographic fingerprints through similarity scoring methods [12].

Recently, metabolomic approaches have been developed which aim to identify and quantify all components within complex mixtures, whereby all of the acquired data can be leveraged to compare phytochemical composition between extracts [13]. However, the ability of metabolomics to encompass the full complexity of phytochemical blends greatly depends on the method used to capture the data. For instance, it has been shown that separative methods provide more accurate statistical models and higher identification rates compared to direct spectroscopic fingerprinting [14]. The choice of analytical tools greatly impacts the final comparative outcomes, as exemplified by the contradictory results obtained for *Serenoa repens* extracts (SrE). Booker and colleagues studied several commercial SrE products using gas chromatography (GC) targeted quantification and 1D-NMR fingerprints. They observed great heterogeneity in the free fatty acid content and found that the extraction solvent used greatly impacted the content of the marketed products [15]. On the other hand, a study by de Combarieu and colleagues also based on comparisons of 1D-NMR fingerprints showed no significant differences between sCO_2_ and hexanic SrE [16]. Due to its low sensitivity, the 1D-NMR technique is a good method to collect a large amount of data and identify most abundant components, but it needs to be complemented by more sensitive approaches, such as liquid chromatography-mass spectrometry (LC-MS) for the detection of minor compounds and to compare the compositions of different samples.

Saw palmetto (*Serenoa repens* (W.Bartram) Small, Arecaceae) is a small palm, growing up to 7-10 feet tall and native to the Southeastern United States. Its dried ripe fruits are yellow and turn black when mature. They were used traditionally by Native Americans to treat genitourinary disturbances [17]. Phytochemical and pharmacological studies of SrE began in the 1870s and focused on lipophilic compounds responsible for the multifactorial efficacy on benign prostatic hyperplasia (BPH), explained mainly by their anti-androgenic, anti-inflammatory and pro-apoptotic effects [18,19]. 

*Serenoa repens* extracts are complex mixtures constituted mainly of free fatty acids (85%) or esterified fatty acids (approximately 2% methyl-ethyl esters and 5–6% triglycerides). The main free fatty acids are lauric (30%), oleic (30%) myristic (10%) and palmitic (10%) acids. Apart from free or esterified fatty acids, triterpenes (1%) and fatty alcohols (0.8 to 1.1%) have also been detected. Other minor components such as polyprenols, carotenoids, tocopherols, hydrocarbons and volatile compounds (1%) have also been described [20,21,22,23,24,25]. *Serenoa repens* extracts are usually analyzed by gas chromatography-mass spectrometry (GC-MS) which involves modifying compounds to aid their detection, including increasing the volatility of fatty acids and the saponification of sterols and other esterified compounds. 

Three types of SrE are commercially available: Hexanic, sCO_2_ and ethanolic extracts, by numerous brands. (See Supplementary Data Table S1). According to the EMA [26], only hexanic SrE are considered as herbal medicinal products for the symptomatic treatment of BPH due to their positive clinical efficacy (well-established use status). Other extracts have traditional use status (ethanolic SrE) or are not listed (sCO_2_ SrE), due to the absence of clinical studies demonstrating their efficacy.

**Figure 1:**
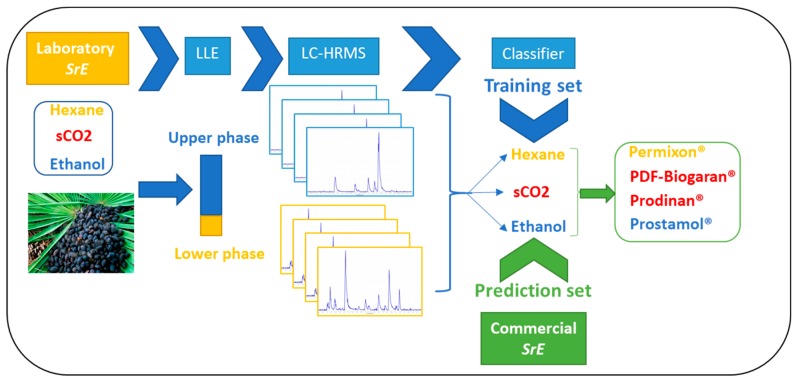
The workflow chart of liquid chromatography-mass spectrometry (LC-MS)-based metabolomic approach: Laboratory and commercial SrE were submitted to a liquid-liquid extraction (LLE) prior to LC-HRMS profiling. A statistical model was set up using laboratory SrE as a training set to classify samples according to their extraction process. Commercial SrE were inserted into the model to predict their respective extraction solvent. Laboratory samples: sCO_2_, supercritical carbon dioxide; EtOH: ethanolic; Hexane: hexanic. Commercial samples: PDF-Biogaran^®^ and Prodinan^®^ (sCO_2_), Prostamol^®^ (EtOH), Permixon^®^ (Hexane).

The aim of this work was to assess the effect of extraction methods on the chemical composition of SrE, using a LC-MS-based metabolomic approach without modification of the native molecules. The objective of this study was to assess the phytochemical composition of hexanic, sCO_2_ or ethanolic-based products. This study was carried out on two sets of samples: Laboratory samples, extracted with the same batch of fruits using the three types of extraction processes under study and, commercial samples, constituted by four brands containing a hexanic SrE (Permixon^®^, Pierre Fabre), two sCO_2_ SrE (Palmier de Floride-PDF-Biogaran^®^, Biogaran and Prodinan^®^, Therabel) and an ethanolic SrE (Prostamol^®^, Menarini). Prior to multivariate data analysis, the chemical compositions of the sample groups were compared. Following this, a supervised model based on laboratory extracts was set up and tested on commercial brands. Finally, the main differences between samples were highlighted (Figure 1).

## 2. Results

### 2.1. Univariate Data Analysis

**Figure 2:**
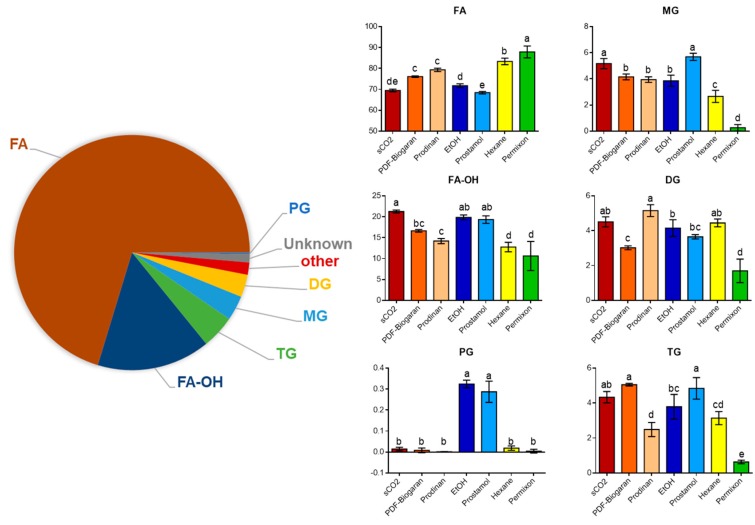
The mean relative content of the main chemical classes for all SrE samples (left, pie chart) and for each sample group (right, bar plots) according to identified features from the LC-MS dataset. One-way ANOVA and Tukey’s post-hoc test (DF = 6.24; *p* ≤ 0.01) were used to assess significant differences among sample groups denoted by a–e letters. FA: free fatty acids; FA-OH: hydroxylated/oxo-fatty acids; MG: Monoglycerides; DG: Diglycerides; TG: Triglycerides; PG: phosphorylated glycerides. Laboratory samples: sCO_2_, supercritical carbon dioxide; EtOH: ethanolic; Hexane: hexanic. Commercial samples: PDF-Biogaran^®^ and Prodinan^®^ (sCO_2_), Prostamol^®^ (EtOH), Permixon^®^ (Hexane).

Our UHPLC-HRMS analysis of 15 laboratory and 20 commercial SrE allowed the annotation of 151 peaks (see Supplementary Table S2), and six chemical classes of lipids accounted for 78% of all annotated peaks (137 peaks). The remaining features belonged to other various chemical classes or could not be attributed (16 unknown peaks). In order to obtain a comprehensive overview of the dataset at the chemical class level, the peak areas of all features belonging to the same class were added together. Although this cannot be considered as a real semi-quantitative reflection of the samples due to compound-dependent ionization intensity and/or matrix effects generated by the ESI source (Electrospray ionization), it provides an overview of the main chemical content of SrE samples (Figure 2, pie chart). As expected, the majority of the compounds detected were free fatty acids and they account for nearly 75% of the mean total ion chromatogram (TIC) area. Oxidized fatty acids and glycerides account for 20% and the remaining parts belong to unidentified features and other chemical classes. A small amount (0.2%) were annotated as glycerophospholipids.

The total area of each lipid class was compared among all sample groups and assessed by one-way ANOVA. Remarkably, some interesting trends could be highlighted (Figure 2, bar plots). On the one hand, the free fatty acid content was significantly higher in hexanic extracts and Permixon^®^ commercial samples. On the other hand, the amount of oxidized fatty acids was significantly lower in these two sample groups compared to the ethanolic and sCO_2_ extracts and other commercial samples. 

Permixon^®^ samples have the lowest content in all glycerides categories. However, sCO_2_ and ethanolic extracts have the highest content of monoglycerides (MG), diglycerides (DG) and triglycerides (TG). Commercial samples displayed some variation. Prodinan^®^ had a high content of DG compared to TG and MG, while PDF-Biogaran^®^ exhibited a higher content of TG compared to DG. Finally, glycerophospholipids were only detected in ethanolic and Prostamol^®^ extracts. 

### 2.2. Multivariate Data Analysis

**Figure 3:**
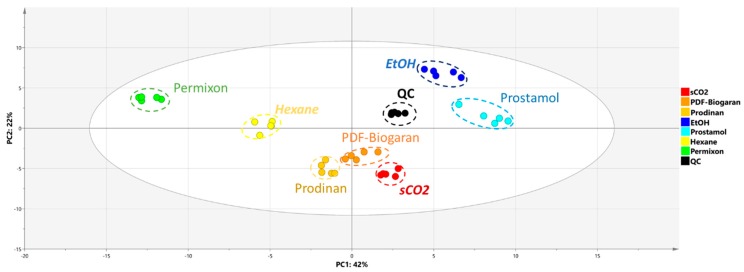
PCA score plot of LC-MS dataset (dotted circles highlight each class for visualization purpose). Laboratory samples: sCO_2_, supercritical carbon dioxide; EtOH: ethanolic; Hexane: hexanic (two superimposed dots observed). Commercial samples: PDF-Biogaran^®^ and Prodinan^®^ (sCO_2_), Prostamol^®^ (EtOH), Permixon^®^ (Hexane). QC: quality control samples.

As a preliminary step in the multivariate analysis, principal component analysis (PCA) was applied as an exploratory data analysis to provide an unsupervised overview of LC-MS fingerprints (Figure 3). Overall, 64% of the total variance was displayed on the first two principal component axes of the PCA score plot. A reproducible response was observed since all independent replicates from the same group clustered together. As expected, quality control samples (QCs) containing an aliquot of each extract were grouped, as they had similar compositions and were located near the center of the plot. It is worth noting that all the clusters are very small in area (Figure 3) and are well split apart in the PCA score plot.

Regarding the first principal component which accounts for 41% of the total explained variance, the main trend seems to be related to the extraction process since ethanolic, sCO_2_ and hexanic extracts are spread across this axis. Interestingly, commercial samples are clustered near laboratory extracts: Prostamol^®^ was close to ethanolic extracts, PDF-Biogaran^®^ and Prodinan^®^ were adjacent to sCO_2_ extracts and Permixon^®^ on the same side as hexanic extracts. 

**Figure 4:**
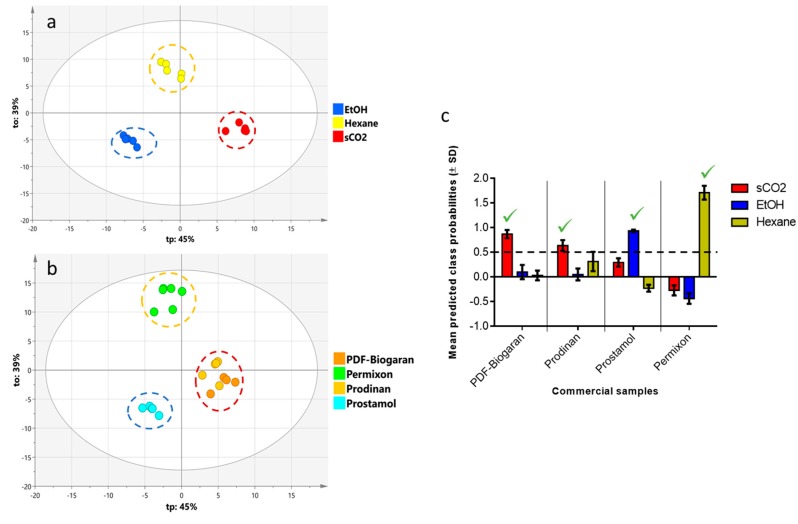
OPLS-DA model based on the solvent extraction process (**a**) the score plot using a laboratory extraction process as the Y class and selected features ranked by ANOVA in X. (**b**) the projection of commercial samples on the same score plot. (**c**) The bar plot of the predicted class probabilities of each commercial sample in their respective extraction process. A value above 0.5 indicates a high level of confidence in the predicted class. Laboratory samples: sCO_2_, supercritical carbon dioxide; EtOH: ethanolic; Hexane: hexanic. Commercial samples: PDF-Biogaran^®^ and Prodinan^®^ (sCO_2_), Prostamol^®^ (EtOH), Permixon^®^ (Hexane).

According to the first unsupervised analysis by PCA in this study, the main driving force involved in the separation of commercial samples seems to be the nature of the extraction solvent. To confirm this hypothesis, the authors filtered features according to their membership to a given extraction process using one-way ANOVA (DF = 2, 13; *p* ≤ 0.01). This ranking procedure allowed the separation of features related to the extraction from other features subject to variation in the preparation of the finished product (i.e. the formulation). As a result, out of 176 features, 89 were retained to build the supervised model (accounting for 52% of all features). The discriminant model using OPLS-DA with the extraction solvent as the Y input and selected features ranked by ANOVA as the X input displayed a good predictive quality (R2Y = 0.977, Q2Y = 0.964; CV-ANOVA = 1.5 × 10^−11^) with a net clustering of laboratory extracts on the score plot (Figure 4a).

To assess the discriminative power of the model, the commercial samples as a prediction set was used, since they were not involved in the construction of the OPLS-DA. Interestingly, Permixon^®^ samples were projected in the same area as hexanic extracts, while PDF-Biogaran^®^ and Prodinan^®^ clustered close to sCO_2_ laboratory extracts. Finally, Prostamol^®^ samples clustered around the ethanolic extracts (Figure 4b). To confirm the ability of the OPLS-DA model to predict the extraction solvent of unknown commercial samples, the Y predictive values were computed for each commercial sample. A value above 0.5 for one class, here the extraction solvent, denotes a high level of confidence in the prediction. The results depicted here showed that PDF-Biogaran^®^ and Prodinan^®^ were extracted using sCO_2,_ while Prostamol^®^ was obtained from an ethanolic extract and Permixon^®^ using hexanic extraction (Figure 4c).

### 2.3. Annotated Biomarkers of SrE Samples

**Figure 5:**
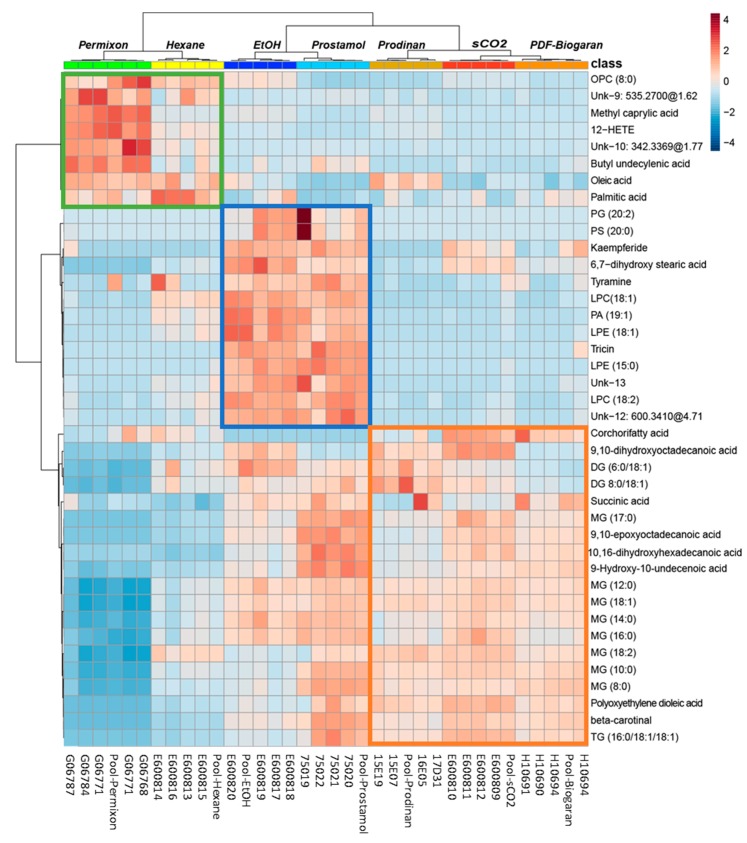
The heatmap combined with a hierarchical cluster analysis of the top 40 VIP ranked features from the OPLS-DA model. Spots are colored according to their peak area in each sample normalized to TIC and Pareto scaled. Colored squares highlight representative features of each extraction process. Laboratory extracts: EtOH: ethanolic; sCO_2_: supercritical carbon dioxide; hexane: hexanic. Commercial samples: Prostamol^®^, PDF-Biogaran^®^, Prodinan^®^, Permixon^®^. OPC (8:0): 10,11-dihydro-12-oxo-15-phytoenoic acid; 12-HETE: 12-Hydroxy-5,8,10,14-eicosatetraenoate; MG: monoglyceride; DG: diglyceride; TG: triglyceride; unk: unknown; PG: Phosphoglyceride; PS: Phosphoserine; LPE: Lysophosphoethanolamine; LPC: Lysophosphatidylcholine. Putative annotations were based on HRMS and MS/MS spectra and *in silico* or experimental matches (See material and methods for details).

According to the OPLS-DA model described above, 52% of the total features detected are directly impacted by the extraction solvent used. To identify the main biomarkers of each extraction process, the 40 top very important parameters (VIP) ranked features from the OPLS-DA model were selected to build a clustered heatmap (Figure 5).

**Figure 6:**
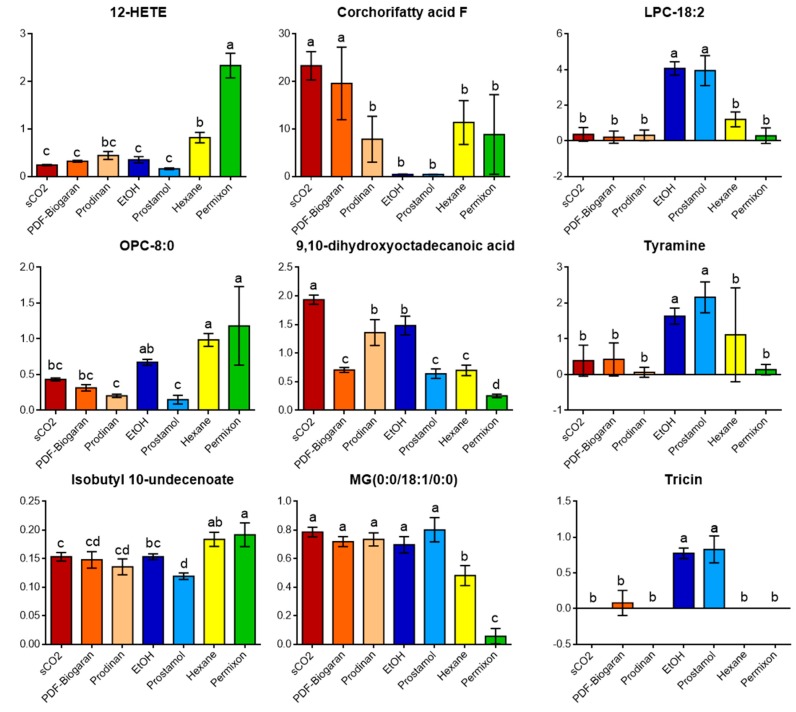
The relative chemical content of annotated biomarkers for each extraction process. The relative mean peak area (±SD) detected in laboratory and commercial samples. One-way ANOVA and Tukey’s post-hoc test (DF = 6.24; *p* ≤ 0.01) were used to assess significant differences among sample groups denoted by a–d letters. Laboratory samples: sCO_2_, supercritical carbon dioxide; EtOH: ethanolic; Hexane: hexanic. Commercial samples: PDF-Biogaran^®^ and Prodinan^®^ (sCO_2_), Prostamol^®^ (EtOH), Permixon^®^ (Hexane). OPC (8:0): 10,11-dihydro-12-oxo-15-phytoenoic acid; 12-HETE: 12-Hydroxy-5,8,10,14-eicosatetraenoate; MG: monoglyceride; LPC: Lysophosphatidlycholine. Putative annotations were based on HRMS and MS/MS spectra and *in silico* or experimental matches (See material and methods for details).

The hierarchical cluster analysis (HCA) used to construct the dendrogram at the top of the heatmap displayed three main clusters. Each cluster corresponds to the laboratory extracts along with their corresponding commercial samples as depicted by the OPLS-DA model. Each category of extracts is well individualized demonstrating a good reproducibility among commercial and laboratory extraction processes. The hexane/Permixon^®^ branch is mainly related to fatty acid compounds like palmitic acid, oleic acid, hydroxylated and esterified fatty acids (12-HETE, methyl caprylate), one oxylipin molecule (OPC 8:0) and two unknown compounds (Unk-9: [M + H]^+^ = 535.2700 × 1.62 min and Unk-10: [M + H]^+^ = 342.3369 × 1.77 min analysed by hydrophilic interaction liquid chromatography (HILIC). Regarding the ethanolic/Prostamol^®^ branch, several phosphoglycerides were highlighted (e.g. PG-20:2/00; LPC-18:1), along with flavonoid derivatives (tricin and kaempferide) and tyramine. Concerning the sCO_2_/Prodinan^®^/PDF-Biogaran^®^ cluster, several glycerolipid compounds were annotated (e.g. MG (18:1), DG (8:0/18:1) and TG (16:0/18:1/18:1)) along with hydroxylated fatty acids (e.g. 9,10-dihydroxyoctadecanoic acid; Corchoryfatty acid F). These two classes of compounds were also detected in the ethanolic/Prostamol^®^ branch which could explain the close proximity of the clusters for ethanolic and sCO_2_ extracts on the PCA score plot (Figure 3). The analysis of variance of individual biomarkers confirmed the heatmap results (Figure 6).

## 3. Discussion

The aim of the present study was to assess the phytochemical composition of SrE from various extraction processes. The LC-MS untargeted metabolomic approach in this study focused on native compounds and allowed the annotation of 151 features of several chemical classes. To the best of the authors knowledge, this study represents the most exhaustive analysis of SrE, as previous works have focused only on certain classes of compounds with extract modification before analysis to aid their detection by GC or LC-APCI-MS (atmospheric pressure chemical ionization), including increasing the volatility of fatty acids or the saponification of sterols and other esterified compounds (fatty acids, sterols, unsaponifiable compounds) [15,16,24]. The choice of the analytical workflow using an ESI source and two orthogonal separative methods (C18 and HILIC) provided a comprehensive overview of SrE chemical content, but was not suitable to detect very long chain fatty acids or esterified sterols. Despite these limitations, significant variations were found in the amounts of the apolar chemical classes between sample groups, with a higher amount of free fatty acids in hexanic and Permixon^®^ samples and a relatively low content of oxidized fatty acids. Interestingly, the authors noticed significant differences in the relative content of esterified fatty acids between laboratory and commercial samples, which could be related to the specific industrial process of each marketed brand. Remarkably, phosphoglycerides are specific to ethanolic and Prostamol^®^ SrE (Figure 2). 

This study used PCA to obtain an overview of the LC-MS fingerprints for SrE which explained 64 % of the total variance in the first two principal components, with PC1 mainly related to the extraction process (Figure 3). Based on this result, a supervised model was set up to discriminate the extraction process. To this end, only compounds common to the extraction process and commercial samples were kept, which accounted for 52% of all the detected features. Interestingly, the OPLS-DA model in this study constructed with laboratory samples was able to predict the extraction process of all the commercial samples. The statistical significance of this model clearly demonstrates that the extraction solvent has an impact on the final composition of SrE (Figure 4). 

The data in this study are in concordance with the unsupervised analysis performed by Booker and colleagues [15], but clearly challenge the conclusions made by the de Combarieu study [16]. Both studies were based on spectroscopic fingerprints using 1D-NMR. Although this method can detect all protonated molecules, its lack of sensitivity means it can only detect the most concentrated metabolites. By contrast, the use of separative methods, such as liquid chromatography, allows us to tune the selectivity which in turn, increases the metabolite coverage. Furthermore, MS-based detection is highly sensitive and can detect compounds in the picomolar range [27]. Here, it was demonstrated that the complementarity of two orthogonal separation modes enables the recognition of several chemical classes, including free and esterified fatty acids, phosphoglycerides and flavonoids (Figure S1). The annotation of metabolites based on high-resolution MS and MS/MS patterns mirrored to *in silico* fragmentation matches or experimental MS/MS library remains hypothetical (level II according to the metabolomics standards initiative) and requires complementary analysis to reach complete identification [28]. Another drawback of MS-based methods is the lack of semi-quantitative data, because in ESI ionization efficiency is structure dependent. However, LC-MS fingerprints are highly reproducible and informative as shown by our data. The multivariate statistical analysis in this study revealed that the extraction solvent has a direct impact on half of the compounds detected.

The lipophilic compounds of SrE responsible for the multifactorial efficacy on BPH explained mainly by their anti-androgenic, anti-inflammatory and pro-apoptotic effects [18,19,22] are partially known. The main components of SrE are fatty acids and numerous published investigations have demonstrated their activities on 5-alpha reductase [29,30]. In vitro studies on 5-alpha reductase have shown important variations among marketed extracts. Permixon^®^ displayed the highest potency, even in batch to batch comparisons. This could be explained by its high fatty acid content [30,31], which was also confirmed in our study. Other compounds of pharmacological interest are partially known, in particular active principles known to have anti-inflammatory effects. Pharmacological activities have also been described for sterols, fatty alcohols and polyprenols isolated from SrE [19,22]. Our results demonstrate heterogeneity among SrE derived using different extraction solvents. For example, ethanolic SrE were enriched in phosphoglycerides, flavonoids and tyramine, whereas hexanic extracts were enriched in OPC (8:0) and 12-HETE (Figure 5 and Figure 6). Tyramine detected in SrE has been implicated in indirect alpha1-adrenoceptor mediated contractions via the release of noradrenaline from sympathetic neurons [32]. However, the pharmacological mechanisms of SrE remain only partially explained [33]. Although in vitro results cannot predict clinical efficacy due to the multifactorial origin of BPH and probable synergistic effects among SrE mixtures, our results emphasize the importance of a thorough method to evaluate the phytochemical composition of SrE, as it could have an impact on clinical efficacy. 

## 4. Materials and Methods

### 4.1. Sample Preparation

Laboratory SrE: Starting from the same batch (batch N° MP24351 collected in Immokalee, FL, USA), dried powdered fruit (10 g) were extracted at room temperature for 2 h using 50 mL *n*-hexane or ethanol 90% (*v*/*v*). After filtration, the filtrate was dried under vacuum. For sCO_2_ extractions, the conditions were as follows: 45 °C, 220 bars, 10 kg CO_2_/h. These processes were performed four times to obtain 12 laboratory SrE. (sCO_2_: E600809, E600810, E600811, E600812, *n*-hexane: E600813, E600814, E600815, E600816, EtOH: E600817, E600818, E600819, E600820).

Commercial SrE: Commercial samples were purchased on the French market as listed below: PDF-Biogaran^®^160 mg (batch N° H10691, H10690, two batches of H10694); Permixon^®^ 160 mg (Batch N° G06784, G06787, G06771, G06768); Prodinan^®^ 160 mg (Batch N° 15E19, 15E07, 17D31, 16E05) and Prostamol^®^ (Batch N° 75020, 75022, 75021, 75019). A dozen capsules content of each of the 16 commercial products were pooled according to each batch number and dissolved in methanol to remove insoluble material and then dried under vacuum to obtain more than 1 g of commercial SrE.

Laboratory and commercial SrE were subject to a liquid-liquid separation starting from 1.00 g of SrE using a mixture of hexane/acetonitrile/water (20 mL/10 mL/5 mL). Hexanic (upper phase) and acetonitrile (lower phase) layers were separated, evaporated to dryness, then solubilized at 2 mg/mL in LC-MS grade methanol (Thermo Fisher Scientific, Hemel Hempstead, UK) for further analyses. A pooled sample of each laboratory extract and commercial SrE groups along with a quality control (QC) sample comprising an aliquot of all SrE was made to monitor the reproducibility of data acquisition. Overall, 36 samples of each phase were analyzed (12 laboratory along with one pool of each extract; 16 commercial samples along with one pool of each group; QC sample). 

### 4.2. Ultra-High-Performance Liquid Chromatography-Orbitrap Analysis

Ultra-high performance liquid chromatography high-resolution mass spectrometry (UHPLC-HRMS) analyses were performed on a UHPLC-DAD-LTQ Orbitrap XL instrument (Ultimate 3000, Thermo Fisher Scientific, Hemel Hempstead, UK). A sample analysis was carried out under positive and negative modes at 15,000 resolving power (full width at half maximum (FWHM) at 400 *m*/*z*). The mass scanning range was *m*/*z* 100–1500, the capillary temperature was 300 °C and the ionization spray voltage was 4.2 kV (positive mode) and 3.0 kV (negative mode). Mass measurement was externally calibrated just before starting the experiment. Each full MS scan was followed by data dependent MS/MS on the four most intense ions using the collision-induced dissociation (CID) fragmentation mode at 35% normalized collision energy, with an isolation width of 2 Da and activation Q set at 0.250. The LC–MS system was run in a binary gradient mode and each sample was injected on two complementary analytical methods to enhance metabolite coverage.

Apolar compounds from both layers were profiled using an Acquity UPLC CSH C18 (100 × 2.1 mm i.d., 1.7 μm, Waters, Milford, MA, USA) column equipped with a guard column. Mobile phase A consisted of water with 20 mM ammonium acetate and mobile phase B was MeOH with 20 mM ammonium acetate. The binary linear solvent gradient was as follows: At 0 min, 25% A-75% B; 12 min, 1% A-99% B; 15 min, 1% A-99% B; 15.5 min, 25% A-75% B; 19 min, 25% A-75% B. The flow rate was 0.3 mL/min, the column temperature was set to 40 °C. The injection volume was 2 µL.

Polar metabolites from lower phases were profiled using a ZIC-pHILIC column (100 × 2.1 mm i.d., 5 µm, SeQuant^®^, Merck, Darmstadt, Germany). Solvent A was 20 mM ammonium acetate and solvent B was acetonitrile; the flow rate was 0.25 mL/min. The gradient was as follows: A 10%-90% B for 0.5 min to A 60%-40% B over 18 min, held at 60% A-40% B for a further 3 min, and then after 0.5 min it returned to initial conditions (A 10%-90% B) and finally held for 5 min for subsequent analysis. The injection volume was 3 µL. 

All samples were injected randomly with one blank and one QC injection at the beginning of the run and then interspersed every six injections.

### 4.3. Data Processing

Raw data from UHPLC-HRMS were processed with MS-DIAL version 3.30 [34] for peak deconvolution and chromatogram alignment. For LC-HRMS/MS, respective MS1 and MS2 tolerance were set to 0.01 and 0.2 Da in centroid mode for an *m*/*z* range between 100 and 1500 Da. The optimized detection threshold was set to 1 × 10^6^ for negative ionization and 2 × 10^6^ for positive ionization concerning MS1 and 50 for MS2 in both ionization modes. Peaks were aligned on a QC reference sample with a retention time tolerance of 0.1 min and a mass tolerance of 0.025 Da and then normalized to total ion chromatogram. Adducts and complexes were excluded from the final peak list. The peak annotation was performed using MS-FINDER 3.12 for the molecular formula (MF) calculation and MS/MS fragmentation pattern matches [35]. The calculation of MF was based on C, H, N, O, P elements and heuristic rules with a mass tolerance of 0.01 Da. Interrogation was firstly based on experimental public MS/MS library and then on *in silico* fragmentation patterns using compound libraries from the Dictionary of Natural Products database (DNP on DVD, v 26.2, CRC press) limited to *Serenoa* genus or Arecaceae family, along with the Human Metabolome Database (HMDB), LIPID MAPS, Chemical Entities of Biological Interest (ChEBI), PlantCyc and KNApSAcK databases included in MS-FINDER. Annotations from the DNP were prioritized when several matches were available. This annotation workflow allowed to reach a level of 2.1 confidence level for experimental library matches and 2.2 for *in silico* based matches [36]. 

The resulting peak lists were then concatenated in both acquisition modes using the R package MScombine [37]. Peak areas from common features detected in both upper and lower phases were added together in the final peak list. Overall, a total of 176 features (*m*/*z* × RT pairs) along with their respective peak areas in each sample were exported in comma-separated value (CSV) format prior to the multivariate data analysis (Annotated data available in Supplementary Table S2).

### 4.4. Statistical Analysis

For multivariate data analysis, CSV files were directly imported into SIMCA-P+ (version 15.0, Sartorius Stedim Biotech, Umetrics, Umeå, Sweden). Data were scaled to unit variance for principal component analysis (PCA). The features mainly related to the extraction solvent were filtered by ANOVA using the variable ranking module in Orange v3.17 [38]. This filtered dataset was Pareto scaled to build the supervised model using orthogonal projection to latent structure-discriminant analysis (OPLS-DA) with extraction solvent as the Y class input and laboratory SrE as X variables. For each model, a leave-one-subject-out cross-validation was performed to assess the model fit. The validity of the discriminant model was verified using permutation tests (Y-scrambling). The predictive capacity of the OPLS-DA model was assessed using commercial SrE as a prediction set. Very important parameter (VIP) scores were used to rank variables according to their correlation with solvent class. Finally, the top 40 ranked features were selected to build a clustered heatmap using Ward distance and the Pearson correlation coefficient with MetaboAnalyst 4.0 [39]. Univariate analyses were performed using PRISM 7.0 (GraphPad, San Diego, CA, USA) for one-way ANOVA and the Tukey post-hoc test. 

## 5. Conclusions

This study examined the phytochemical composition of SrE, using a multiplexed metabolomic approach for the first time on native compounds. To the best of the authors knowledge, this study represents the most exhaustive analysis of SrE. This study observed significant differences in the chemical classes of fatty acids between sample groups, with a higher amount of free fatty acids in hexanic and Permixon^®^ samples and a relatively low content of oxidized fatty acids. The multivariate statistical analysis revealed that the extraction solvent has a direct impact on half of the compounds detected. This metabolomics study unequivocally demonstrates that sCO_2_, hexanic and ethanolic extracts are not chemically equivalent. The results in this study highlighted significant differences in major and minor compounds between the three extracts and the related finished products. The chemical profile of fatty acids alone is insufficient to assess the similarity between sCO_2_ and hexanic SrE. Minor compounds should also be considered when evaluating pharmacological efficacy and toxicological safety. The analysis in this study suggests dramatic differences in product blends according to the type of extraction procedure carried out. These differences will eventually impact clinical outcomes. 

## Supplementary Materials

The following are available online, Figure S1: LC-MS chromatogram (QC sample) in negative and positive ESI modes using C18 or HILIC separation columns, Table S1: Extraction Method of the Top 10 *Serenoa repens* Medicinal Products Sold in Europe, Table S2: Annotated LC-MS dataset.
