# Supplementary material for: Comparison of the Phytochemical Composition of Serenoa repens Extracts by a Multiplexed Metabolomic Approach
Source: Molecules. 2019 Jun 13;24(12):2208. doi: 10.3390/molecules24122208 (PMC6631273; doi:10.3390/molecules24122208)
Supplement: Supplementary file 1 [file molecules-24-02208-s001.zip › Revised Table S1.docx]

| **Extraction Method of the Top 10 Serenoa Repens Medicinal Products Sold in Europe (1)** | | | |
| --- | --- | --- | --- |
| **Brand Name(s)** | **Extraction Method** | **Year of Launch (2)** | **Laboratory**  **(Home Country)** |
| **PERMIXON** | **Hexane** | **1982** | **Pierre Fabre (France)** |
| **PROSTAMOL** | **EtOH90% (v/v)** | **1997** | **Menarini**  **(Italy)** |
| **PROSTAGUTT-PROSTAKAN-UROGUTT-**  **PROSTAPLANT (3)** | **EtOH90% (v/v)** | **1991** | **Schwabe**  **(Germany)** |
| **PROSTA URGENIN** | **EtOH90% (v/v)** | **1993** | **Meda**  **(Sweden)** |
| **GRANU FINK PROSTA (4)** | **EtOH90%(v/v)** | **1999** | **Perrigo**  **(Ireland)** |
| **SABALLO** | **EtOH96% (v/v)** | **NA** | **Teva**  **(Israel)** |
| **PRODINAN-PROSTASERENE** | **SC-CO2** | **1993** | **Therabel**  **(France)** |
| **PROSTASAN** | **EtOH96% (v/v)** | **1969** | **Bioforce**  **(Switzerland)** |
| **PALMIER DE FLORIDE BIOGARAN** | **SC-CO2** | **2018** | **Biogaran**  **(France)** |
| **PROSTESS** | **EtOH96% (v/v)** | **1992** | **TAD**  **(Germany)** |
| *(1) Source: IQVIA Customer Insight MAT/6/18 – Ranking in volume sold (Standard Units)*  *(2) In the first country of launch*  *(3) Serenoa Repens is usually associated with Urtica Dioica*  *(4) Serenoa Repens is associated with Cucurbita Pepo*  ***In blue color: products that are commercialized in France*** | | | |
