# Supplementary material for: Comparison of the Phytochemical Composition of Serenoa repens Extracts by a Multiplexed Metabolomic Approach
Source: Molecules. 2019 Jun 13;24(12):2208. doi: 10.3390/molecules24122208 (PMC6631273; doi:10.3390/molecules24122208)
Supplement: Supplementary file 1 [file molecules-24-02208-s001.zip › Figure S1-Molecules.pptx]

## Slide 1
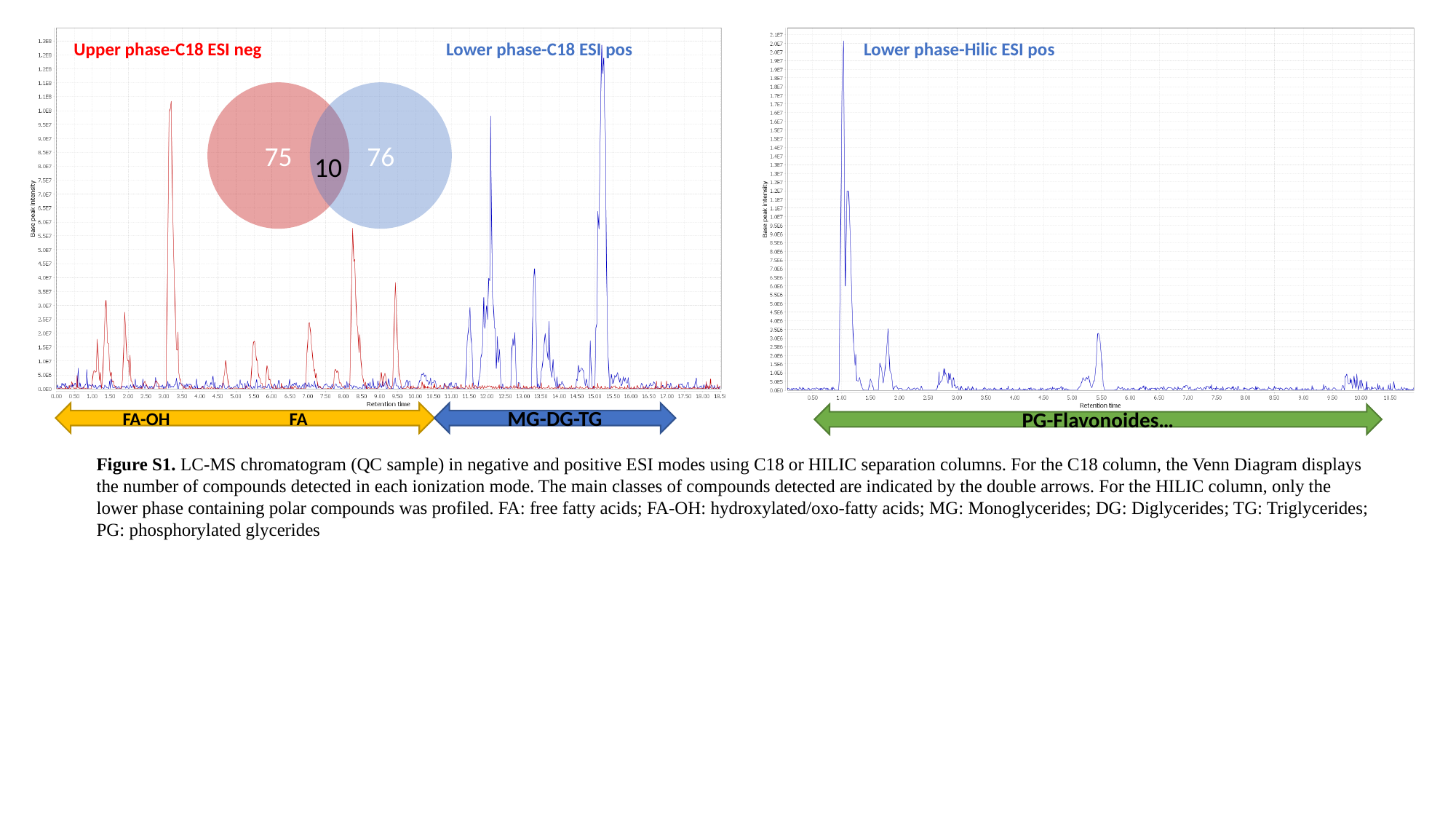

Lower phase-C18 ESI pos
Lower phase-Hilic ESI pos
Upper phase-C18 ESI neg
75
76
10
FA
FA-OH
MG-DG-TG
PG-Flavonoides…
Figure S1. LC-MS chromatogram (QC sample) in negative and positive ESI modes using C18 or HILIC separation columns. For the C18 column, the Venn Diagram displays the number of compounds detected in each ionization mode. The main classes of compounds detected are indicated by the double arrows. For the HILIC column, only the lower phase containing polar compounds was profiled. FA: free fatty acids; FA-OH: hydroxylated/oxo-fatty acids; MG: Monoglycerides; DG: Diglycerides; TG: Triglycerides; PG: phosphorylated glycerides
